# Worse histopathology and prognosis in women with breast cancer diagnosed during the second trimester of pregnancy

**DOI:** 10.1016/j.esmoop.2024.102972

**Published:** 2024-03-22

**Authors:** L. Gkekos, F.E. Lundberg, K. Humphreys, I. Fredriksson, A.L.V. Johansson

**Affiliations:** 1 Department of Medical Epidemiology and Biostatistics, Karolinska Institutet, Stockholm, Sweden; 2 Department of Oncology-Pathology, Karolinska Institutet, Stockholm, Sweden; 3 Department of Molecular Medicine and Surgery, Karolinska Institutet, Stockholm, Sweden; 4 Department of Breast, Endocrine Tumors and Sarcoma, Karolinska University Hospital, Stockholm, Sweden; 5 Cancer Registry of Norway, Norwegian Institute of Public Health, Oslo, Norway

**Keywords:** pregnancy, survival, breast cancer, subtype, trimester, population based

## Abstract

**Background:**

Evidence suggests that women with breast cancer diagnosed during pregnancy (PrBC) and within 2 years of delivery (PPBC) have similar survival compared to women diagnosed not near pregnancy if adjusted for stage and subtype. To investigate whether this is true for all subtypes and for both pregnancy and post-delivery periods, we examined clinicopathologic features and survival in women with breast cancer by trimesters and 6-month post-delivery intervals.

**Materials and methods:**

Women diagnosed with invasive breast cancer during 1992-2018 at ages 18-44 years were identified in the Swedish Cancer Register, with information on childbirths from the Swedish Multi-Generation Register and clinical data from Breast Cancer Quality Registers. Each woman with PrBC or PPBC was matched 1 : 2 by age and year to comparators diagnosed with breast cancer not near pregnancy. Distributions of stage, grade, and surrogate subtypes were compared. Adjusted hazard ratios (HRs) with 95% confidence intervals (CIs) for breast cancer mortality were estimated using Cox regression.

**Results:**

We identified 1430 women with PrBC and PPBC (181 during pregnancy, 499 during the first and 750 during the second year after delivery). Compared to 2860 comparators, women with PrBC and PPBC in the first year after delivery had a significantly higher proportion of luminal human epidermal growth factor receptor 2 (HER2)-positive, HER2-positive and triple-negative tumours, and more advanced stage at diagnosis. After adjustment for age, year, parity, country of birth, hospital region, subtype, and stage, women diagnosed during the second trimester had a worse prognosis than matched comparators (HR 1.8, 95% CI: 1.0-3.2).

**Conclusions:**

Women diagnosed during pregnancy or within the first year after delivery have a worse prognosis than women diagnosed not near pregnancy due to adverse tumour biology and advanced stage at diagnosis. A worse prognosis for women diagnosed during the second trimester remained after multivariable adjustment, possibly reflecting difficulties to provide optimal treatment during ongoing pregnancy.

## Highlights

• Breast cancer in pregnancy had higher proportions of luminal HER2-positive, HER2-positive, and triple-negative subtypes.
• Women diagnosed in the second and third trimesters, and first year after delivery had significantly worse prognosis.
• The worse prognosis remained significant only for the second trimester after adjusting for tumour biology and stage.

## Introduction

Breast cancer is the most commonly diagnosed form of cancer in pregnant or recently pregnant women with an estimated incidence of 17.5 to 39.9 per 100 000 births (during pregnancy and 1 year after delivery). This incidence is increasing in many populations.^1^^,^^2^ Pregnancy-associated breast cancer (PABC) is often defined as breast cancer diagnosed during pregnancy (PrBC) and 2 years after delivery (postpartum breast cancer, PPBC). Since diagnostic procedures and treatments differ according to whether the cancer is diagnosed during pregnancy or after delivery, the breast cancer diagnosed during pregnancy should be assessed separately from post-delivery cases.^3^ Current international guidelines state that women diagnosed with PrBC should be given breast cancer treatment that resembles that in nonpregnant young women to the greatest possible extent, depending on tumour characteristics and trimester of pregnancy.^4^, ^5^, ^6^

Published evidence shows that women diagnosed with PABC have a poorer prognosis compared to women with breast cancer not near pregnancy.^7^, ^8^, ^9^, ^10^, ^11^ A recent meta-analysis reported a 60% increased mortality in women diagnosed during pregnancy and within 12 months of delivery.^12^ Importantly, several studies have found no significant associations between diagnosis during pregnancy and prognosis,^13^, ^14^, ^15^, ^16^, ^17^, ^18^ after adjusting for tumour biology and stage, implying that pregnancy in itself may not carry an excess risk beyond that of poorer tumour characteristics if adequate treatment is given.^4^ The adverse tumour characteristics, i.e. estrogen receptor (ER)-negative, human epidermal growth factor receptor 2 (HER2)-positive disease, and advanced stage, could be related to a suppression of certain tumours during pregnancy and lactation due to pregnancy-related physiological changes and immunomodulation.^19^ The advanced stage observed in PABC could also be due to diagnostic delays following misinterpretation of cancer signs and symptoms coinciding with increased breast tissue density.^20^^,^^21^

Although pregnancy-related effects and treatment options vary across trimesters and post-delivery periods, the majority of published studies on prognosis do not make a distinction between trimester and post-delivery time windows and instead assume a constant risk across pregnancy. Only two studies have investigated the separate effects of trimesters on survival and indicate a higher mortality after diagnosis in the second and third trimesters.^9^^,^^22^

We have previously reported on the tumour characteristics and prognosis of PABC in Sweden.^17^ In this update, which includes an additional 8 years of diagnoses and follow-up (652 additional patients), we aimed to assess tumour characteristics of PrBC and PPBC by trimesters and post-delivery intervals, and subsequent impact on breast cancer survival. All comparisons were made to matched comparators diagnosed with breast cancer not near pregnancy, to assess whether pregnancy carries increased risk for adverse outcomes. We also assessed whether prognosis in women with PrBC and PPBC differs by age, year, breast cancer subtype, stage, and follow-up time in order to identify high-risk groups.

## Materials and methods

### Study population

In this population-based matched cohort study, data from several Swedish registers were individually linked via the personal identification number assigned to all residents of Sweden. The Multi-Generation Register (MGR) is based on the total population register and allows for linkage of any individual born from 1932 and onwards with their parents (enabling identification of family structures).^23^ The Medical Birth Register (MBR) includes all live- and stillbirths in Sweden after a pregnancy of at least 28 weeks (during 1973-2008) and at least 22 weeks (after 2008). The Swedish Cancer Register (SCR) includes all incident cases of cancer in Sweden coded according to the current version of the International Classification of Diseases (ICD) and is back-translated to ICD version 7.

The study cohort was defined as all women registered in the MGR that had a diagnosis of invasive breast cancer in SCR between 1992 and 2018 at ages 18-44 years. For each woman, we included the first occurrence of a breast cancer diagnosis (ICD-10 code C50; *n* = 15 183). In the presence of more than one invasive breast cancer diagnosis within 90 days of the first one, the tumour with the largest size and most nodal involvement was included (*n* = 348 women, 2.2%). Sixteen women (*n* = 16) were excluded due to inconsistent emigration history (women who emigrated before receiving their diagnosis). In total, 15 171 women were included in the cohort.

Detailed clinical breast cancer information was linked to the cohort from the historical Regional Breast Cancer Quality Registers (BCQR) 1992-2007 (*n* = 7242) and the Swedish National Quality Register for Breast Cancer (NKBC) 2008-2017 (*n* = 6592), the latter of which has very high completeness with a coverage across regions and years of >99%.^24^ A matching criterion of −30/+90 days difference in the recorded diagnosis dates in the SCR and the breast cancer registers was used. A matching record in the breast cancer registers was not found for 1337 (8.8%) women. Information was obtained on ER (</≥10%) and progesterone receptor (PR) status (</≥10% for BCQR, </≥20% for NKBC), HER2 status, and grade. We also included clinical and pathological TNM (tumour–node–metastasis) stage based on the definition by the Union for International Cancer Control.^25^ Treatment information was obtained on surgery (breast conserving surgery, mastectomy, neither/other), chemotherapy, radiotherapy, endocrine therapy, and anti-HER2 treatment.

Breast cancer surrogate subtypes were defined as luminal A-like (ER+/PR+/HER2−/grade I-II or ER−/PR+/HER2−/grade I-II), luminal B-like (ER+/PR+/HER2−/grade III or ER+/PR−/HER2−/ grade I-III or ER−/PR+/HER2−/grade III), luminal HER2-positive (ER+/PR+/HER2+/ grade I-III or ER+/PR−/HER2+/ grade I-III), HER2-positive (ER−/PR+/HER2+/ grade I-III or ER−/PR−/HER2+/ grade I-III), and triple-negative breast cancer (TNBC; ER−/PR−/HER2−/grade I-III) (Supplementary Table S1, available at https://doi.org/10.1016/j.esmoop.2024.102972).

### Exposure to pregnancy and after delivery periods and matching of comparators

Women were considered exposed (PrBC or PPBC) if they received a breast cancer diagnosis during pregnancy or within 24 months after delivery, based on delivery date from MGR and breast cancer diagnosis date from SCR. In case of multiple pregnancies, within the 2-year window, the pregnancy closest to the breast cancer diagnosis was chosen. Women that received a breast cancer diagnosis outside this time window were considered unexposed (non-PABC). If conception date was available (defined by last menstrual period, ultrasound, or *in vitro* fertilisation) in MBR, timing of diagnosis was defined by trimesters (first: 0-97 days; second: 98-188 days; third: >188 days). If conception date was not available (0.1%), trimesters were defined using delivery date (first: 280-183 days, second: 182-92 days; third: 91-0 days before the delivery date).

To eliminate confounding by age and year at diagnosis, women diagnosed with breast cancer before pregnancy, >2 years after delivery, or in nulliparous women (comparators) were randomly selected in a ratio of 1 : 2 to the women with PrBC or PPBC within matching strata by age at diagnosis (18-29, 30-34, 35-36, 37-38, 39-40, 41-42, 43-44), year of diagnosis (1992-2007, 2008-2018), and information availability from the quality registers (BCQR, NKBC, and neither). The final matched cohort consisted of 1430 women with PrBC and PPBC matched to 2860 comparator women. Strata with identical values for the matching variables were pooled together to increase power.

Information on maternal migration dates and country of birth was obtained from the total population register and educational level from the LISA database at Statistics Sweden. Information on the date and cause of death was acquired from the Swedish Cause of Death Register.

### Statistical analysis

Since several important covariates were incomplete, multiple imputation with chained reactions (MICE) was applied to reduce bias and increase power of the analysis (Supplementary Methods, available at https://doi.org/10.1016/j.esmoop.2024.102972). Each variable was imputed conditional on all other variables over 61 cycles to generate as many datasets on which the regression model was applied. The resulting parameter estimates and standard errors were pooled together using Rubin’s rule to yield one inference.

Based on the matched sample, frequencies and proportions of tumour characteristics across pre- and post-delivery intervals were calculated based on complete data and were compared using chi-square tests of association. Similarly, proportions were estimated from the imputed datasets based on logistic regression models for binary outcomes and multinomial logistic regression for categorical outcomes. The pooled estimates of proportions of tumour characteristics in the exposed group were compared to the proportions among matched comparators using Wald tests (Supplementary Table S2, available at https://doi.org/10.1016/j.esmoop.2024.102972). Tumour characteristics were compared for PrBC and PPBC (first and second year after delivery) separately, as well as by finer exposure groups (first trimester, second trimester, third trimester, and post-delivery intervals 0-3, 4-6, 7-9, 10-12, 13-18 and 19-24 months).

In the survival analysis, follow-up was defined from the date of diagnosis until death due to breast cancer or censoring. Censoring events included first emigration after diagnosis, death due to other causes, another cancer diagnosis, end of study (31 December 2018), and completion of 15 years of follow-up, whichever came first. Cause-specific survival was estimated using the Kaplan–Meier method and stratified by finer exposure groups (first trimester, second trimester, third trimester, or by post-delivery intervals 0-6, 7-12, 13-18 and 19-24 months after delivery, as well as matched comparators) and compared using Wilcoxon tests. Breast cancer mortality rates were estimated as the number of deaths over person-time at risk.

The association between PrBC and PPBC exposure groups and the breast cancer mortality rate among the women was modelled with stratified Cox proportional hazard regression models yielding hazard ratios (HRs) with 95% confidence intervals (CIs). The models were stratified on the matching variables and further adjusted for year of diagnosis (1992-1999, 2000-2004, 2005-2009, 2010-2014, 2015-2018), parity before diagnosis (no previous children, 1 child, 2 children, 3+ children), health care region (Stockholm-Gotland, Uppsala-Örebro, Southeast, South, West, North), country of birth (Nordic, non-Nordic), breast cancer subtype, and stage. Associations with exposure groups were assessed with Wald tests. Interactions between exposure groups and age, year, subtype and stage, as well as with follow-up (non-proportional hazards) were assessed using F tests suggested by Li et al. (1991) implemented in mi test in Stata.^26^^,^^27^ Due to quality issues, treatment variables were only included in the main analysis as auxiliary variables in the multiple imputation. A sensitivity analysis was carried out including additional adjustment for treatment.

All tests were two-sided and *P* values of <0.05 were considered statistically significant. SAS version 9.4 was used for data management (SAS Institute Inc., Cary, NC) and Stata 17 software for statistical analyses (StataCorp. 2021. Stata Statistical Software: Release 17. College Station, TX: StataCorp LLC).

Ethical approvals for the study were obtained from the Ethical Review Board in Stockholm (DNR: 2010-1950-31/4; 2011-599-32, 2018/1293-32) and the Swedish Ethical Review Authority (DNR: 2022-02992-02).

## Results

### Background characteristics

The median length of follow-up in the cohort was 7.4 years (PrBC: 6.8 years, comparators: 7.7 years; PPBC: 7.1 years, comparators: 7.6 years). Of women diagnosed during pregnancy, 25 (13.8%) were diagnosed during the first, 59 (32.6%) during the second, and 123 (53.6%) during the third trimester. Women with PrBC were younger compared to those with PPBC, especially in the second year after delivery (Supplementary Table S3, available at https://doi.org/10.1016/j.esmoop.2024.102972). Compared to matched comparators, women with PrBC and PPBC had lower parity and a higher educational level, while there were no large differences in proportion being born outside the Nordic countries.

### Tumour characteristics

Compared to matched comparators, women diagnosed with PrBC had significantly larger tumours, while ER and PR negativity were also significantly more prominent (Table 1). Among ER-negative tumours (both PrBC and PPBC) we observed 13 (0.9%) with low ER expression (1%-9%) among those exposed and 10 (0.3%) among the matched comparators. Grade among PrBC only differed significantly in the observed data (*P* value = 0.021), and not in the imputed (*P* value = 0.096). Lastly, the subtype distribution differed significantly between PrBC and the matched comparators, with luminal HER2-positive, HER2-positive, and TNBC tumours being more frequent in women with PrBC.

**Table 1:** Tumour characteristics of women diagnosed with breast cancer during pregnancy and within 2 years after delivery compared to matched comparators, Sweden 1992-2018

	During pregnancy		First year after delivery		Second year after delivery	
	Matched comparators *n* (%)	PrBC *n* (%)	Matched comparators *n* (%)	PPBC *n* (%)	Matched comparators *n* (%)	PPBC *n* (%)
Total no. of observations	362	181	998	499	1500	750
Tumour size (T)						
T1	128 (37.9)	37 (21.8)	385 (41.8)	99 (21.6)	545 (39.5)	266 (38.5)
T2	171 (50.6)	87 (51.2)	409 (44.5)	256 (55.9)	654 (47.4)	324 (46.9)
T3	39 (11.5)	46 (27.1)	126 (13.7)	103 (22.5)	180 (13.1)	101 (14.6)
Missing	24	11	78	41	121	59
*P* value^a^		<0.001		<0.001		0.612
*P* value^b^		<0.001		<0.001		0.620
Lymph nodal involvement (N)^a^						
N0	178 (52.0)	86 (50.3)	469 (51.1)	183 (40.0)	695 (50.4)	296 (42.8)
N1	124 (36.3)	48 (28.1)	312 (34.0)	173 (37.8)	479 (34.7)	281 (40.7)
N2	33 (9.6)	22 (12.9)	105 (11.4)	61 (13.3)	148 (10.7)	79 (11.4)
N3	7 (2.0)	15 (8.8)	32 (3.5)	41 (9.0)	58 (4.2)	35 (5.1)
Missing	20	10	80	41	120	59
*P* value^a^		0.001		<0.001		0.012
*P* value^b^		0.004		<0.001		0.015
Distant metastasis (M)^a^						
M0	312 (97.8)	155 (97.5)	847 (98.0)	414 (94.5)	1272 (98.8)	641 (98.0)
M1	7 (2.2)	4 (2.5)	17 (2.0)	24 (5.5)	16 (1.2)	13 (2.0)
Missing	43	22	134	61	212	96
*P* value^a^		0.825		0.001		0.200
*P* value^b^		0.774		0.001		0.322
Stage^a^						
Stage 0 + I	81 (25.6)	24 (15.1)	238 (27.7)	54 (12.4)	326 (25.6)	137 (21.1)
Stage II	111 (35.1)	58 (36.5)	290 (33.8)	141 (32.3)	444 (34.8)	225 (34.7)
Stage III	117 (37.0)	73 (45.9)	314 (36.6)	218 (49.9)	489 (38.4)	273 (42.1)
Stage IV	7 (2.2)	4 (2.5)	17 (2.0)	24 (5.5)	16 (1.3)	13 (2.0)
Missing	46	22	139	62	225	102
*P* value^a^		0.058		<0.001		0.084
*P* value^b^		0.104		<0.001		0.102
ER status^a^						
Negative	72 (30.6)	64 (52.9)	175 (28.5)	131 (45.5)	261 (26.4)	153 (31.5)
Positive	163 (69.4)	57 (47.1)	438 (71.5)	157 (54.5)	727 (73.6)	332 (68.5)
Missing	127	60	385	211	512	265
*P* value^a^		<0.001		<0.001		0.040
*P* value^b^		<0.001		<0.001		0.016
PR status^a^						
Negative	93 (40.4)	66 (54.1)	211 (34.8)	169 (59.7)	380 (38.8)	209 (44.1)
Positive	137 (59.6)	56 (45.9)	396 (65.2)	114 (40.3)	600 (61.2)	265 (55.9)
Missing	132	59	391	216	520	276
*P* value^a^		0.014		<0.001		0.053
*P* value^b^		0.006		<0.001		0.008
HER2 status^a^						
Negative	136 (67.0)	70 (68.0)	405 (73.1)	173 (64.1)	639 (75.9)	293 (69.3)
Positive	67 (33.0)	33 (32.0)	149 (26.9)	97 (35.9)	203 (24.1)	130 (30.7)
Missing	159	78	444	229	658	327
*P* value^a^		0.865		0.008		0.012
*P* value^b^		0.677		0.001		0.009
Nottingham grade^a^						
Grade I	14 (6.8)	4 (3.7)	56 (9.7)	13 (5.1)	105 (12.1)	20 (4.8)
Grade II	74 (35.9)	25 (23.1)	190 (33.0)	61 (24.1)	322 (37.2)	159 (37.9)
Grade III	118 (57.3)	79 (73.1)	330 (57.3)	179 (70.8)	439 (50.7)	240 (57.3)
Missing	156	73	422	246	634	331
*P* value^a^		0.021		0.001		<0.001
*P* value^b^		0.096		0.001		<0.001
Surrogate subtype^a^						
Luminal A-like	38 (26.4)	14 (20.3)	129 (33.7)	22 (13.3)	236 (38.7)	76 (26.8)
Luminal B-like	37 (25.7)	13 (18.8)	102 (26.6)	43 (26.1)	147 (24.1)	76 (26.8)
Luminal HER2 positive	31 (21.5)	15 (21.7)	70 (18.3)	32 (19.4)	95 (15.6)	66 (23.2)
HER2 positive	11 (7.6)	8 (11.6)	25 (6.5)	21 (12.7)	36 (5.9)	15 (5.3)
TNBC	27 (18.8)	19 (27.5)	57 (14.9)	47 (28.5)	96 (15.7)	51 (18.0)
Missing	218	112	615	334	890	466
*P* value^a^		0.389		<0.001		0.004
*P* value^b^		0.021		<0.001		0.001

ER, estrogen receptor; HER2, human epidermal growth factor receptor 2; PR, progesterone receptor; PPBC, breast cancer diagnosed after delivery; PrBC, breast cancer diagnosed during pregnancy; TNBC, triple-negative breast cancer.

a Chi-square test of association based on complete case data.

b Wald test based on imputed data. Post-estimated counts and proportions are presented in Supplementary Table S2, available at https://doi.org/10.1016/j.esmoop.2024.102972.

Women with PPBC presented more often with lymph nodal metastases, while only those diagnosed during the first year after delivery had significantly larger tumours and significantly more distant metastases. Women with PPBC had more prominent ER and PR negativity as well as a significantly higher proportion of HER2-positive tumours compared to their matched comparators. Lastly, the subtype distribution differed significantly between PPBC and their matched comparators, with the luminal HER2-positive, HER2-positive, and TNBC subtypes being more frequent in women with PPBC.

**Figure 1:**
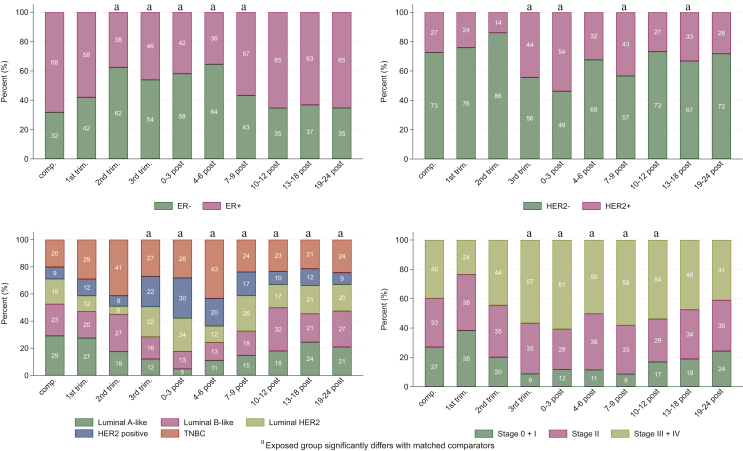
Distribution of tumour characteristics in women diagnosed with breast cancer during pregnancy and within 2 years after delivery in Sweden in 1992-2018, compared to in-matched comparators by trimesters and post-delivery periods. comp., matched comparators; trim., trimester; post, months post-delivery. ER, estrogen receptor; HER2, human epidermal growth factor receptor 2; TNBC, triple-negative breast cancer.

Secondly, we subdivided women with PrBC by trimesters and PPBC by post-delivery periods at diagnosis. No significant differences in tumour biology or stage were found for PrBC diagnosed in the first trimester and comparators. Compared to matched comparators, tumours diagnosed during the second trimester were significantly more often ER negative, while tumours diagnosed during the third trimester were significantly larger, more often had distant metastases, were ER and PR negative, and of luminal HER2-positive, HER2-positive or TNBC subtypes (Figure 1; Supplementary Figure S1 and Table S4, available at https://doi.org/10.1016/j.esmoop.2024.102972). Similarly, significant associations between stage and ER, PR, HER2 status, and subtype were found in all 3-month periods 0-9 months after delivery, and for stage, PR, grade, and subtype during 10-12 months after delivery. Associations with lymph nodal involvement, PR, grade, and subtype were also observed in the tumour diagnosed 19-24 months after delivery. Only lymph nodal involvement was significantly different in the earlier period (13-18 months after delivery).

As expected, women with PrBC underwent more mastectomy and received more chemotherapy and less endocrine therapy compared to comparators (Supplementary Table S5, available at https://doi.org/10.1016/j.esmoop.2024.102972). In contrast, women with PPBC more often underwent mastectomy, had higher proportions of chemotherapy (if diagnosed within 1 year after delivery) and more anti-HER2 therapy.

### Breast cancer mortality of PrBC and PPBC compared to comparators

Among 181 women with PrBC, 45 died due to breast cancer during the 15 years of follow-up, while 133 and 156, respectively, died among women with PPBC in the first and second year after delivery (Table 2). The cause-specific mortality rate among women diagnosed during pregnancy was 36.4 (95% CI: 27.2-48.7) per 1000 person-years, while during the first year after delivery it was 39.4 (95% CI: 33.2-46.7), and during the second year after delivery it was 28.3 (95% CI: 24.2-33.1).

**Table 2:** Associations between pre- and post-delivery diagnosis and breast cancer death in women aged 18-44 years in Sweden between 1992 and 2018

	Number of breast cancers	Number of deaths	Mortality rate per 1000 person-years (95% CI)	Model 1 HR^a^ (95% CI)	Model 2 HR^a^ (95% CI)	Model 3 HR^a^ (95% CI)
Matched comparators	2860	528	19.1 (18.4-19.9)	1.0 (ref)	1.0 (ref)	1.0 (ref)
Women with PrBC or PPBC						
PrBC—during pregnancy	181	45	36.4 (27.2-48.7)	1.8 (1.2-2.7)	1.7 (1.1-2.5)	1.5 (1.0-2.3)
PPBC—first year after delivery	499	133	39.4 (33.2-46.7)	1.6 (1.2-2.0)	1.4 (1.1-1.8)	1.2 (0.9-1.5)
PPBC—second year after delivery	750	156	28.3 (24.2-33.1)	1.1 (0.9-1.4)	1.1 (0.9-1.3)	1.0 (0.8-1.3)
*P* value^b^				<0.001	<0.001	<0.001
PrBC—first trimester	25	3	18.6 (6.0-57.8)	1.0 (0.3-3.3)	1.0 (0.3-3.3)	1.3 (0.4-4.5)
PrBC—second trimester	59	18	42.0 (26.5-66.7)	2.0 (1.2-3.5)	1.8 (1.1-3.2)	1.8 (1.0-3.2)
PrBC—third trimester	97	24	37.1 (24.9-55.3)	1.8 (1.1-3.0)	1.7 (1.1-2.8)	1.4 (0.8-2.3)
PPBC—0-6 months after delivery	154	39	37.8 (27.6-51.8)	1.4 (1.0-2.0)	1.3 (0.9-1.8)	1.1 (0.8-1.6)
PPBC—7-12 months after delivery	345	94	40.0 (32.7-49.0)	1.6 (1.3-2.1)	1.5 (1.2-2.0)	1.2 (0.9-1.6)
PPBC—13-18 months after delivery	375	79	28.2 (22.6-35.2)	1.1 (0.8-1.4)	1.0 (0.8-1.4)	1.0 (0.8-1.3)
PPBC—19-24 months after delivery	375	77	28.3 (22.7-35.4)	1.2 (0.9-1.5)	1.1 (0.9-1.4)	1.1 (0.9-1.5)
*P* value^b^				<0.001	<0.001	<0.001

CI, confidence interval; HR, hazard rate ratio; PPBC, breast cancer diagnosed after delivery; PrBC, breast cancer diagnosed during pregnancy.

a HRs from Cox regression models (separate models were fitted for the broad and finer PrBC/PPBC exposure groups) on imputed datasets that were pooled using Rubin’s rules. Model 1: Adjusted for matching variables (age at diagnosis, year of diagnosis, quality register information availability), parity, country of birth and health care region. Model 2: Same as model 1 and further adjusted for breast cancer subtypes. Model 3: Same as model 2 and further adjusted for tumour stage.

b Test based on imputed data.

Women with PrBC or PPBC during the first year after delivery experienced significantly higher breast cancer mortality (HR 1.8, 95% CI: 1.2-2.7 and 1.6, 1.2-2.0, respectively) compared to their matched comparators after adjustment for background characteristics. The association remained after adjusting for breast cancer subtypes (HR 1.7, 95% CI: 1.1-2.5 and 1.4, 1.1-1.8 respectively). After additional adjustment for stage, HRs were further reduced and only remained significant in women diagnosed during pregnancy (HR 1.5, 95% CI: 1.0-2.3, *P* value = 0.045).

**Figure 2:**
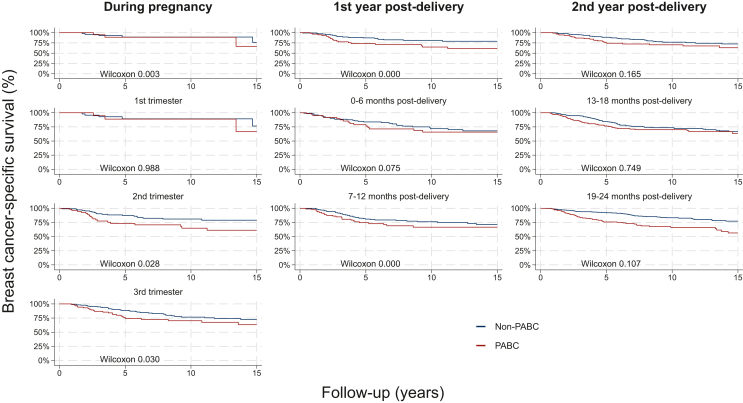
Kaplan–Meier curves for 15-year breast cancer-specific survival for PABC versus their matched non-PABC comparators. PABC, pregnancy-associated breast cancer.

When subdividing by trimesters and post-delivery periods, the survival proportions were significantly lower in women diagnosed during the third trimester and 10-12 months after delivery compared to age- and year-matched comparators (Figure 2). In the Cox regression analysis, women diagnosed during the second trimester had significantly higher breast cancer mortality rates after adjustment for subtype and stage (HR 1.8, 95% CI: 1.0-3.2, *P* value = 0.036) (Table 2). Women diagnosed during the third trimester had significantly higher breast cancer mortality rates after adjustment for confounding factors and subtype (HR 1.7, 95% CI: 1.1-2.8), yet the association was no longer significant after further adjustment for stage (HR 1.4, 95% CI: 0.8-2.3). We found no association if the woman was diagnosed in the first trimester, although the number of events was low. In the post-delivery window, diagnosis of PPBC during the 7-12 months after delivery was associated with a 40% higher mortality rate (HR 1.5, 95% CI: 1.2-2.0) after adjusting for subtype. However, the association was no longer present after adjusting for stage (HR 1.2, 95% CI: 0.9-1.6).

Overall, the association between PABC and breast cancer mortality was consistent across age groups, breast cancer subtypes, and stages with non-significant tests of interaction (Table 3). However, compared to the matched comparators, increased risks of death were found in women who were 40-44 years of age and diagnosed during the second year after delivery, and women with a stage 0 or stage I tumour diagnosed during pregnancy or the first year after delivery. There was a significant interaction with follow-up, with higher mortality rates within the first 5 years of follow-up if the women were diagnosed with breast cancer during pregnancy or during the first year after delivery (*P* value = 0.013).

**Table 3:** Associations between PrBC and PPBC, and breast cancer death by age, year, and subtype in women aged 18-44 years in Sweden during 1992-2018

	Matched comparators	PrBC	PPBC—first year after delivery	PPBC—second year after delivery
	HR (95% CI)	HR (95% CI)	HR (95% CI)	HR (95% CI)
Overall	1.0 (ref)	1.5 (1.0-2.3)	1.2 (0.9-1.5)	1.0 (0.8-1.3)
Age at diagnosis, years				
18-29	1.0 (ref)	1.7 (0.6-4.9)	1.5 (0.8-2.7)	1.3 (0.7-2.4)
30-34	1.0 (ref)	1.5 (0.8-3.0)	1.2 (0.9-1.8)	0.8 (0.6-1.2)
35-39	1.0 (ref)	1.4 (0.7-2.8)	0.9 (0.6-1.4)	1.0 (0.7-1.4)
40-44	1.0 (ref)	1.6 (0.5-5.4)	1.4 (0.7-2.7)	1.5 (1.0-2.4)
*P* value^a^ = 0.656				
Breast cancer subtype				
Luminal A-like	1.0 (ref)	1.0 (0.2-5.0)	1.2 (0.6-2.4)	1.2 (0.7-2.0)
Luminal B-like	1.0 (ref)	2.1 (0.9-4.9)	1.2 (0.7-2.0)	0.9 (0.5-1.4)
Luminal HER2 positive	1.0 (ref)	1.2 (0.4-3.9)	1.3 (0.7-2.5)	1.2 (0.7-2.1)
HER2 positive	1.0 (ref)	1.0 (0.3-3.4)	1.1 (0.6-2.0)	1.1 (0.5-2.2)
TNBC	1.0 (ref)	1.7 (0.9-3.5)	1.1 (0.7-1.8)	1.0 (0.6-1.6)
*P* value^a^ = 0.992				
TNM stage				
Stage 0 + I	1.0 (ref)	2.8 (1.1-7.1)	2.6 (1.3-5.3)	1.1 (0.6-2.1)
Stage II	1.0 (ref)	1.6 (0.8-3.1)	1.1 (0.7-1.7)	0.8 (0.6-1.3)
Stage III	1.0 (ref)	1.5 (0.9-2.4)	1.1 (0.8-1.5)	1.1 (0.8-1.4)
Stage IV	1.0 (ref)	0.4 (0.1-3.5)	1.0 (0.5-2.2)	1.6 (0.6-4.4)
*P* value^a^ = 0.355				
Follow-up, years				
<5	1.0 (ref)	2.0 (1.2-3.2)	1.5 (1.1-2.0)	1.2 (0.9-1.6)
≥5	1.0 (ref)	0.9 (0.4-2.0)	0.8 (0.5-1.2)	0.9 (0.6-1.2)
*P* value^a^ = 0.013				

Models estimated separately for each interaction between exposure status and age at diagnosis, subtype, TNM stage, and follow-up, respectively, with adjustment for matching variables (age at diagnosis, year of diagnosis, quality register information availability), parity, country of birth, health care region, breast cancer subtype, and tumour stage.

CI, confidence interval; HER2, human epidermal growth factor receptor 2; HR, hazard ratio; PPBC, breast cancer diagnosed post-delivery; PrBC, breast cancer diagnosed during pregnancy; TNBC, triple-negative breast cancer; TNM, tumour–node–metastasis.

a Test for interactions based on imputed data.

In sensitivity analyses, further adjusting for treatment did not change the estimates (Supplementary Table S6, available at https://doi.org/10.1016/j.esmoop.2024.102972).

## Discussion

In this large population-based study, women diagnosed with breast cancer during pregnancy or within 2 years after delivery exhibited more aggressive tumour biology and more advanced stage compared to those diagnosed not near pregnancy, in particular if diagnosed during the third trimester or within 9 months after delivery. In addition, we found a higher breast cancer mortality in women diagnosed during the second and third trimesters and during the first year after delivery. This association was fully explained by the adverse tumour biology and stage if the woman was diagnosed in the third trimester or after delivery, but not in the second trimester where the association remained after adjustments. The higher mortality in women with PrBC or PPBC during the first year after delivery was confined to the first 5 years of follow-up, after which mortality was comparable to women diagnosed not near pregnancy. This is in line with a recent meta-analysis showing an overall increased mortality for PABC patients who persisted up to 5 years after diagnosis.^12^

### Breast cancer during pregnancy

Our findings corroborate several studies showing that women with PrBC more often have aggressive tumours in terms of ER and PR negativity,^7^^,^^10^^,^^11^^,^^13^^,^^17^ HER2 positivity,^7^^,^^15^ larger tumour size,^9^^,^^10^^,^^14^^,^^17^ lymph nodal involvement,^10^^,^^11^^,^^17^^,^^28^ distant metastases,^11^ more advanced stage,^7^, ^8^, ^9^^,^^13^^,^^17^ higher-grade tumours,^7^^,^^10^^,^^11^^,^^13^ and of HER2-positive or TNBC subtypes.^7^^,^^8^^,^^13^

Our finding of larger tumour size and more advanced stage in the third trimester likely reflects both subtype-specific growth patterns and a possible delay in diagnosis of these tumours. Both HER2-expressing/amplified subtypes (luminal HER2 positive and HER2 positive) were more frequent in those diagnosed during the third trimester. The proportion of HER2 positivity at diagnosis in the second trimester, on the other hand, did not differ from that among women diagnosed not near pregnancy. There was a significant shift towards more ER-negative tumours diagnosed during the second trimester where around half were ER negative, confirming existing literature.^9^^,^^29^

The reasons for more adverse tumour characteristics during pregnancy and post-delivery periods are largely unknown. One hypothesis is that the estrogen surge during pregnancy promotes the proliferation of breast cancer stem-like cells, which has been reported to be mostly ER negative.^30^^,^^31^ This could also relate to a suppression of certain tumours during pregnancy and lactation due to pregnancy-related immunomodulation.^19^ The pregnancy-related physiological changes of the breast, such as hypertrophy and increased breast tissue density, could also mask the tumour in diagnostic examinations as well as the patient’s perception of symptoms.^20^^,^^21^

As estrogen is the main driver of the histological changes of the breast during pregnancy,^32^ the increased proportion of hormone-independent tumours in the later trimesters could reflect an indirect effect of estrogen on promoting tumour growth independent of ER,^33^ and a potential pro-oncogenic transition of existing ER/PR-negative breast cancer stem-like cells.^29^, ^30^, ^31^

Importantly, we found a higher breast cancer mortality in women diagnosed during the second and third trimesters, which after adjustments for tumour biology and stage remained significant only in the second trimester. Treatment in the second trimester is challenging, especially if the tumour is presenting more aggressive characteristics, while the selection of treatment options in the third trimester is broader. Despite our estimates remaining essentially unchanged after adjustment for treatment, we cannot exclude residual confounding by treatment due to the lower quality of our treatment information, especially in the earlier study period. In addition, residual confounding by tumour biology and stage may be present as women diagnosed during pregnancy may have presented with more adverse tumour characteristics also within levels of these characteristics. Only two studies have previously investigated trimester-specific effects, with varying results. Dimitrakakis et al. (2013) found a worse prognosis during the third trimester while, similar to our findings, Suelmann et al. (2022) found a worse prognosis during the second trimester, which remained in multivariable analysis.^9^^,^^22^

### Breast cancer after delivery

Our results show that women diagnosed in the first year after delivery more often had adverse tumour characteristics, e.g. advanced stage, ER negativity, higher grade, and more often HER2-positive and TNBC subtypes than women diagnosed not near pregnancy, with stronger associations within the first 9 months of delivery. Stage distribution was comparable to women diagnosed during the third trimester, but with worse subtype distribution. Reasons for these findings could be lactation-related factors such as tissue density, diagnostic delay, or tumour-promoting tissue remodelling relating to the involution after weaning.^34^ Although we were unable to assess differences by lactation status, a recent study by Suelmann et al., 2022 found that a post-delivery diagnosis in lactating individuals was associated with less aggressive tumours compared to PrBC diagnosed in the third trimester.^9^ However, the effect of lactation is difficult to separate from pregnancy itself, since pregnancy leads to maturation of breast tissue and mammographic density, and involution effects persist several months after weaning. In Sweden, >85% of babies are breastfed at 2 months, and around 50%-70% at 6 months.^35^

Women diagnosed during the first year after delivery had an increased breast cancer mortality, especially if diagnosed 7-12 months after delivery. However, the association was not present after adjustment for subtype and stage. Hence, the association can be fully explained by the adverse tumour characteristics, and recent pregnancy or lactation in itself does not carry an excess risk. Thus, provided that these women are given treatment according to guidelines, they have the same survival as women diagnosed not near pregnancy with similar tumour characteristics. Only a few studies have investigated tumour characteristics separately for the post-delivery period. We and others have found a higher proportion of ER-negative tumours among women diagnosed with PPBC,^9^^,^^17^^,^^36^ and some studies have also found worse prognosis after adjustments for this group.^10^^,^^17^^,^^37^

Diagnosis during the second year after delivery had no impact on breast cancer survival and there were only minor differences in tumour characteristics when compared to women diagnosed not near pregnancy. Diagnostic delay due to previous pregnancy is unlikely to occur in the second year after delivery, as around 80% of Swedish women have finished breastfeeding within a year and the breast tissue is in the process of gradually returning to its pre-pregnancy state.^35^

This is the first study to date evaluating breast cancer characteristics and survival by trimesters and finer post-delivery intervals compared to carefully matched comparator women of similar age and year of diagnosis. The population-based setting, with extensive clinical data and complete follow-up, provided unbiased ascertainment of both cancers and pregnancies, avoiding the selection bias that is common in institutional-based studies. This study also included well-matched population-based comparators with the same level of high-quality data. By not matching on tumour biology and stage, this study avoided overmatching, and enabled the investigation of histopathologic markers in PABC tumours and their impact on cancer survival. In addition, traditional ways of handling missing data, such as carrying out complete case analysis, were not used as they may have introduced bias or may have led to loss of efficiency. Instead, we used multiple imputation, which allowed us to circumvent these limitations, and to investigate subgroups to a greater extent than has been done in previous studies.

The most important limitation of the study was the lack of information on pregnancy terminations in the form of miscarriages or abortions before 22^+0^ weeks of gestation (or 28^+0^ before July 2008), which resulted in an under-ascertainment of PrBC cases in the first trimester. A recent study from Denmark showed that 84% of PrBC in the first trimester resulted in an abortion.^11^ Similarly, a Dutch study found that diagnoses in the first trimester accounted for 33.3% of all PrBC in comparison to the 13.8% in our study.^9^ However, for the second and third trimesters, the numbers in our study are in line with both these previous studies. Our study also lacked data regarding breastfeeding and whether the tumours were detected through screening mammography invitations, which starts at age 40 in Sweden. Furthermore, although we had some information on the given treatment, we did not have access to detailed treatment information, e.g. type of chemotherapy received or the timing of it.

In conclusion, we found a significantly higher breast cancer mortality in women diagnosed during pregnancy and within 9 months after delivery, which remained only for those diagnosed during the second trimester after adjustment for subtype and tumour stage. Although one cannot rule out insufficient adjustment for tumour biology, it seems plausible that treatment difficulties during ongoing pregnancy might explain this effect on survival. Our results underline the importance of no treatment delays and chemotherapy dose adjustments due to pregnancy to optimise treatment results in this high-risk group.
