# Supplementary material for: Worse histopathology and prognosis in women with breast cancer diagnosed during the second trimester of pregnancy
Source: ESMO Open. 2024 Mar 22;9(4):102972. doi: 10.1016/j.esmoop.2024.102972 (PMC10980937; doi:10.1016/j.esmoop.2024.102972)
Supplement: Supplementary Tables [file mmc1.docx]

**Supplemental Tables & Figure**

| **Supplemental Table S1: Surrogate subtypes definition** | | | | |
| --- | --- | --- | --- | --- |
| **ER** | **PR** | **HER2** | **Grade** | **Surrogate subtypes** |
| Positive | Positive | Positive | Any | Luminal HER2 positive |
| Positive | Positive | Negative | I | Luminal A-like |
|  |  |  | II | Luminal A-like |
|  |  |  | III | Luminal B-like |
| Positive | Negative | Positive | Any | Luminal HER2 positive |
| Positive | Negative | Negative | Any | Luminal B-like |
| Negative | Positive | Positive | Any | HER2 positive |
| Negative | Positive | Negative | I | Luminal A-like |
|  |  |  | II | Luminal A-like |
|  |  |  | III | Luminal B-like |
| Negative | Negative | Positive | Any | HER2 positive |
| Negative | Negative | Negative | Any | TNBC |
| ER; oestrogen receptor, PR; progesterone receptor, HER2; human epidermal growth factor receptor 2 | | | | |

| **Supplemental Table S2. Tumour characteristics of women diagnosed with breast cancer during pregnancy and 2 years post-delivery 1992-2018.** | | | | | | |
| --- | --- | --- | --- | --- | --- | --- |
|  | **During pregnancy** | | **1^st^ year post-delivery** | | **2^nd^ year post-delivery** | |
|  | **Matched**  **comparators**  **N (%)** | **PrBC**  **N (%)** | **Matched**  **comparators**  **N (%)** | **PPBC**  **N (%)** | **Matched**  **comparators**  **N (%)** | **PPBC**  **N (%)** |
| **Total no. of observations** | 362 | 181 | 998 | 499 | 1500 | 750 |
| **Tumour size (T)^a^** |  |  |  |  |  |  |
| T1 | 138 (38.2) | 40 (21.9) | 413 (41.4) | 106 (21.3) | 588 (39.2) | 285 (38.0) |
| T2 | 182 (50.3) | 92 (51.1) | 447 (44.8) | 279 (55.9) | 713 (47.5) | 354 (47.2) |
| T3 | 42 (11.6) | 49 (27.0) | 138 (13.9) | 114 (22.8) | 199 (13.3) | 111 (14.8) |
| *p-value*^b^ |  | <0.001 |  | <0.001 |  | 0.620 |
| **Lymph nodal involvement (N) ^a^** |  |  |  |  |  |  |
| N0 | 188 (52.0) | 91 (50.1) | 504 (50.5) | 197 (39.4) | 750 (50.0) | 319 (42.6) |
| N1 | 131 (36.1) | 51 (28.1) | 342 (34.3) | 188 (37.6) | 521 (34.7) | 303 (40.4) |
| N2 | 35 (9.7) | 23 (13.0) | 116 (11.7) | 68 (13.6) | 165 (11.0) | 88 (11.7) |
| N3 | 8 (2.1) | 16 (8.8) | 36 (3.6) | 47 (9.4) | 65 (4.3) | 40 (5.3) |
| *p-value*^b^ |  | 0.004 |  | <0.001 |  | 0.015 |
| **Distant metastasis (M) ^a^** |  |  |  |  |  |  |
| M0 | 354 (97.9) | 177 (97.5) | 978 (98.0) | 471 (94.4) | 1479 (98.6) | 735 (98.0) |
| M1 | 8 (2.1) | 4 (2.5) | 20 (2.0) | 28 (5.6) | 21 (1.4) | 15 (2.0) |
| *p-value*^b^ |  | 0.774 |  | 0.001 |  | 0.322 |
| **Stage ^a^** |  |  |  |  |  |  |
| Stage 0 + I | 93 (25.6) | 30 (16.5) | 277 (27.8) | 63 (12.7) | 387 (25.8) | 162 (21.6) |
| Stage II | 128 (35.3) | 64 (35.4) | 332 (33.2) | 159 (31.9) | 519 (34.6) | 256 (34.2) |
| Stage III | 134 (37.0) | 83 (45.6) | 369 (37.0) | 248 (49.8) | 574 (38.2) | 317 (42.3) |
| Stage IV | 8 (2.1) | 4 (2.5) | 20 (2.0) | 28 (5.6) | 21 (1.4) | 15 (2.0) |
| *p-value*^b^ |  | 0.104 |  | <0.001 |  | 0.102 |
| **ER status ^a^** |  |  |  |  |  |  |
| Negative | 119 (32.8) | 100 (55.0) | 309 (31.0) | 228 (45.7) | 446 (29.7) | 268 (35.8) |
| Positive | 243 (67.2) | 81 (45.0) | 689 (69.0) | 271 (54.3) | 1054 (70.3) | 482 (64.2) |
| *p-value*^b^ |  | <0.001 |  | <0.001 |  | 0.016 |
| **PR status ^a^** |  |  |  |  |  |  |
| Negative | 154 (42.5) | 103 (57.1) | 387 (38.8) | 305 (61.1) | 614 (40.9) | 360 (48.0) |
| Positive | 208 (57.5) | 78 (42.9) | 611 (61.2) | 194 (38.9) | 886 (59.1) | 390 (52.0) |
| *p-value*^b^ |  | 0.006 |  | <0.001 |  | 0.008 |
| **HER2 status ^a^** |  |  |  |  |  |  |
| Negative | 255 (70.4) | 123 (68.2) | 739 (74.1) | 317 (63.5) | 1136 (75.7) | 519 (69.3) |
| Positive | 107 (29.6) | 58 (31.8) | 259 (25.9) | 182 (36.5) | 364 (24.3) | 231 (30.7) |
| *p-value*^b^ |  | 0.677 |  | 0.001 |  | 0.009 |
| **Grade ^a^** |  |  |  |  |  |  |
| Grade I | 30 (8.3) | 8 (4.5) | 103 (10.3) | 29 (5.8) | 174 (11.6) | 36 (4.8) |
| Grade II | 126 (34.8) | 44 (24.3) | 330 (33.1) | 122 (24.5) | 540 (36.0) | 273 (36.4) |
| Grade III | 206 (56.8) | 129 (71.2) | 565 (56.6) | 348 (69.7) | 787 (52.4) | 441 (58.8) |
| *p-value*^b^ |  | 0.096 |  | 0.001 |  | <0.001 |
| **Surrogate subtype ^a^** |  |  |  |  |  |  |
| Luminal A-like | 99 (27.4) | 29 (16.1) | 294 (29.5) | 70 (14.1) | 486 (32.4) | 170 (22.7) |
| Luminal B-like | 79 (21.8) | 37 (20.4) | 252 (25.3) | 109 (21.9) | 362 (24.2) | 179 (23.8) |
| Luminal HER2 positive | 72 (20.0) | 28 (15.5) | 177 (17.8) | 99 (19.9) | 244 (16.3) | 151 (20.1) |
| HER2 positive | 35 (9.6) | 30 (16.4) | 81 (8.2) | 83 (16.7) | 120 (8.0) | 79 (10.6) |
| TNBC | 77 (21.2) | 57 (31.7) | 193 (19.3) | 137 (27.5) | 288 (19.2) | 171 (22.8) |
| *p-value*^b^ |  | 0.021 |  | <0.001 |  | 0.001 |
| PrBC; Breast cancer diagnosed during pregnancy, PPBC; Breast cancer diagnosed post-delivery, ER; oestrogen receptor, PR; progesterone receptor, HER2; human epidermal growth factor receptor 2  **^a^** Frequencies modelled from logistic and multinomial regressions to utilise the imputed datasets  ^b^ Wald test based on imputed data. | | | | | | |

| **Supplemental Table S3. Background characteristics of women diagnosed with breast cancer during pregnancy and 2 years post-delivery 1992-2018.** | | | | | | |
| --- | --- | --- | --- | --- | --- | --- |
|  | **During pregnancy** | | **1^st^ year post-delivery** | | **2^nd^ year post-delivery** | |
|  | **Matched comparators**  **N (%)** | **PrBC**  **N (%)** | **Matched comparators**  **N (%)** | **PPBC**  **N (%)** | **Matched comparators**  **N (%)** | **PPBC**  **N (%)** |
| **Total no. of observations** | 362 | 181 | 998 | 499 | 1500 | 750 |
| **Age at diagnosis** |  |  |  |  |  |  |
| 18-24 years | 9 (2.5) | 1 (0.6) | 18 (1.8) | 3 (0.6) | 20 (1.3) | 7 (0.9) |
| 25-29 years | 57 (15.7) | 32 (17.7) | 110 (11.0) | 61 (12.2) | 96 (6.4) | 51 (6.8) |
| 30-34 years | 144 (39.8) | 72 (39.8) | 372 (37.3) | 186 (37.3) | 482 (32.1) | 241 (32.1) |
| 35-39 years | 122 (33.7) | 59 (32.6) | 346 (34.7) | 179 (35.9) | 575 (38.3) | 293 (39.1) |
| 40-44 years | 30 (8.3) | 17 (9.4) | 152 (15.2) | 70 (14.0) | 327 (21.8) | 158 (21.1) |
| *p-value* **^a^** |  | 0.568 |  | 0.366 |  | 0.912 |
| **Calendar year at diagnosis** |  |  |  |  |  |  |
| 1992-1999 | 79 (21.8) | 33 (18.2) | 209 (20.9) | 96 (19.2) | 353 (23.5) | 176 (23.5) |
| 2000-2004 | 58 (16.0) | 27 (14.9) | 163 (16.3) | 64 (12.8) | 227 (15.1) | 87 (11.6) |
| 2005-2009 | 62 (17.1) | 40 (22.1) | 179 (17.9) | 115 (23.0) | 265 (17.7) | 154 (20.5) |
| 2010-2014 | 82 (22.7) | 40 (22.1) | 249 (24.9) | 137 (27.5) | 341 (22.7) | 174 (23.2) |
| 2015-2018 | 81 (22.4) | 41 (22.7) | 198 (19.8) | 87 (17.4) | 314 (20.9) | 159 (21.2) |
| *p-value* **^a^** |  | 0.652 |  | 0.052 |  | 0.150 |
| **Parity prior to diagnosis** |  |  |  |  |  |  |
| No previous children | 124 (34.3) | 66 (36.5) | 339 (34.0) | 154 (30.9) | 421 (28.1) | 207 (27.6) |
| 1 child | 67 (18.5) | 69 (38.1) | 154 (15.4) | 201 (40.3) | 236 (15.7) | 344 (45.9) |
| 2 children | 126 (34.8) | 31 (17.1) | 401 (40.2) | 96 (19.2) | 603 (40.2) | 147 (19.6) |
| 3+ children | 45 (12.4) | 15 (8.3) | 104 (10.4) | 48 (9.6) | 240 (16.0) | 52 (6.9) |
| *p-value* **^a^** |  | <0.001 |  | <0.001 |  | <0.001 |
| **Highest achieved education** |  |  |  |  |  |  |
| ≤9 years | 21 (5.8) | 14 (7.7) | 86 (8.6) | 37 (7.4) | 119 (7.9) | 45 (6.0) |
| 10-13 years | 176 (48.6) | 53 (29.3) | 425 (42.6) | 173 (34.7) | 620 (41.3) | 254 (33.9) |
| 13-14 years | 47 (13.0) | 28 (15.5) | 158 (15.8) | 83 (16.6) | 252 (16.8) | 127 (16.9) |
| >14 years | 116 (32.0) | 83 (45.9) | 324 (32.5) | 204 (40.9) | 499 (33.3) | 318 (42.4) |
| Missing | 2 (0.6) | 3 (1.7) | 5 (0.5) | 2 (0.4) | 10 (0.7) | 6 (0.8) |
| *p-value* **^a^** |  | <0.001 |  | 0.006 |  | <0.001 |
| **Country of birth** |  |  |  |  |  |  |
| Nordic countries | 284 (78.5) | 147 (81.2) | 815 (81.7) | 399 (80.0) | 1226 (81.7) | 605 (80.7) |
| Other countries | 78 (21.5) | 34 (18.8) | 183 (18.3) | 100 (20.0) | 274 (18.3) | 145 (19.3) |
| *p-value* **^a^** |  | 0.453 |  | 0.427 |  | 0.540 |
| **Healthcare region** |  |  |  |  |  |  |
| Stockholm-Gotland | 99 (27.3) | 66 (36.5) | 287 (28.8) | 147 (29.5) | 395 (26.3) | 208 (27.7) |
| Uppsala-Örebro | 70 (19.3) | 22 (12.2) | 189 (18.9) | 80 (16.0) | 292 (19.5) | 107 (14.3) |
| South-east | 37 (10.2) | 18 (9.9) | 99 (9.9) | 51 (10.2) | 153 (10.2) | 79 (10.5) |
| South | 60 (16.6) | 26 (14.4) | 183 (18.3) | 78 (15.6) | 253 (16.9) | 149 (19.9) |
| West | 68 (18.8) | 35 (19.3) | 168 (16.8) | 104 (20.8) | 294 (19.6) | 162 (21.6) |
| North | 28 (7.7) | 14 (7.7) | 72 (7.2) | 39 (7.8) | 113 (7.5) | 45 (6.0) |
| *p-value* **^a^** |  | 0.019 |  | 0.292 |  | 0.021 |
| PrBC; Breast cancer diagnosed during pregnancy, PPBC; Breast cancer diagnosed post-delivery  **^a^** Chi-squared test of association based on complete case data | | | | | | |

**
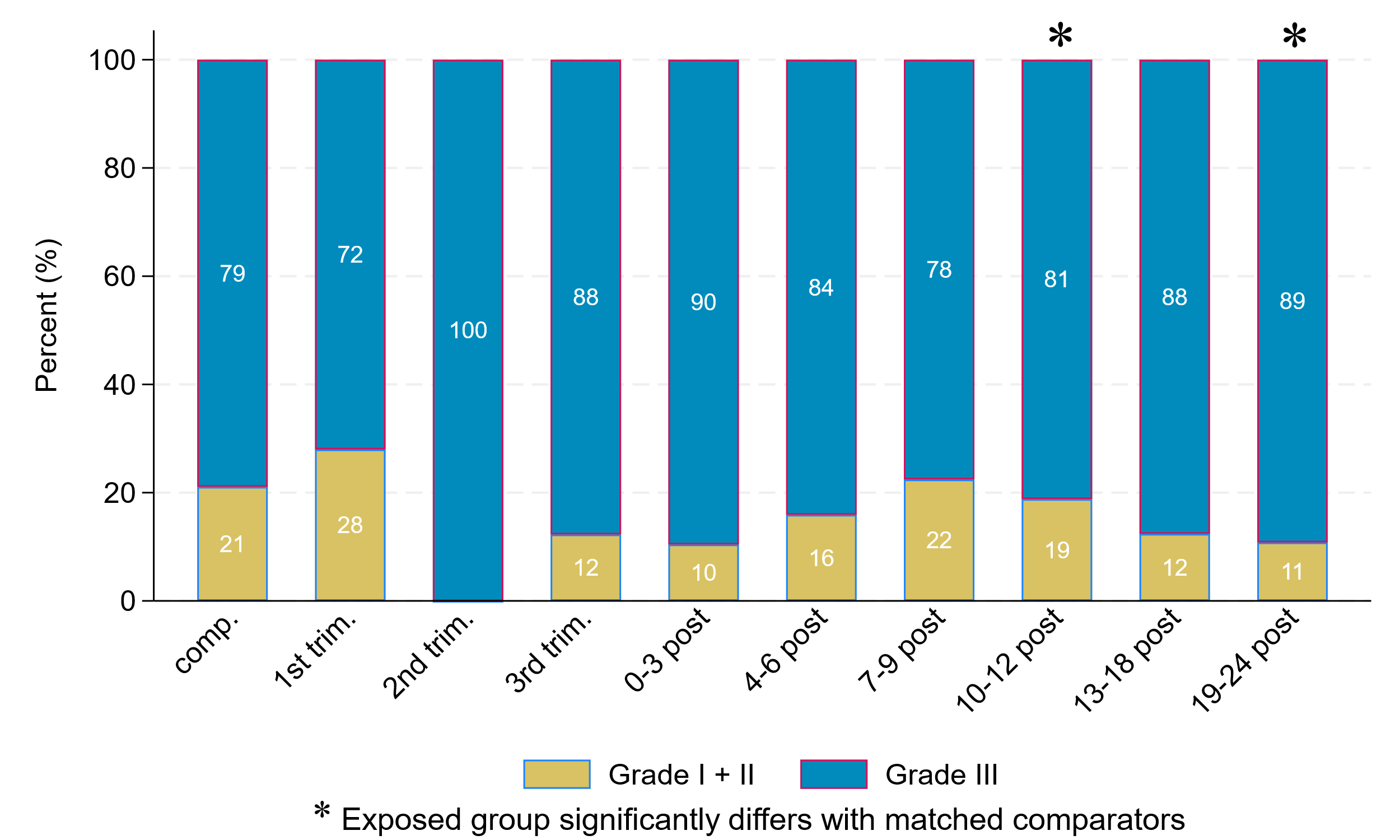
Supplemental Figure S1. Distribution of Grade in women diagnosed with breast cancer during pregnancy and within 2 years post-delivery in Sweden 1992-2018, compared to in matched comparators by trimesters and post-delivery periods.**

**Comp.; Matched comparators, trim.; trimester, post; months post-delivery**

| **Supplemental Table S4. Tumour characteristics of women diagnosed with breast cancer during pregnancy and within 2 years post-delivery compared to matched controls by trimesters and post-delivery periods, Sweden 1992-2018.** | | | | | | | | | | | | | | | | | | | | | | | | | | |
| --- | --- | --- | --- | --- | --- | --- | --- | --- | --- | --- | --- | --- | --- | --- | --- | --- | --- | --- | --- | --- | --- | --- | --- | --- | --- | --- |
|  | **1^st^ trimester** | | **2^nd^ trimester** | | | **3^rd^ trimester** | | | **0-3 months post-delivery** | | | **4-6 months post-delivery** | | | **7-9 months post-delivery** | | | **10-12 months post-delivery** | | | **13-18 months post-delivery** | | | **19-24 months post-delivery** | | |
|  | **comp.**  **N (%)** | **PrBC**  **N (%)** | **comp.**  **N (%)** | **PrBC**  **N (%)** | **comp.**  **N (%)** | | **PrBC**  **N (%)** | **comp.N (%)** | | **PPBC**  **N (%)** | **comp.**  **N (%)** | | **PPBC**  **N (%)** | **comp.**  **N (%)** | | **PPBC**  **N (%)** | **comp.**  **N (%)** | | **PPBC**  **N (%)** | **comp.**  **N (%)** | | **PPBC**  **N (%)** | **comp.**  **N (%)** | | **PPBC**  **N (%)** |  |
| **Total no. of observations** | 50 | 25 | 118 | 59 | 194 | | 97 | 132 | | 66 | 176 | | 88 | 308 | | 154 | 382 | | 191 | 750 | | 375 | 750 | | 375 |  |
| **Tumour size (T)** |  |  |  |  |  | |  |  | |  |  | |  |  | |  |  | |  |  | |  |  | |  |  |
| T1 | 18 (39.1) | 10 (43.5) | 40 (37.4) | 14 (25.9) | 70 (37.8) | | 13 (14.0) | 61 (50.4) | | 9 (15.5) | 55 (35.3) | | 13 (16.3) | 111 (39.8) | | 23 (16.7) | 158 (43.4) | | 54 (29.7) | 251 (36.5) | | 116 (33.6) | 294 (42.5) | | 150 (43.4) |  |
| T2 | 22 (47.8) | 11 (47.8) | 54 (50.5) | 27 (50.0) | 95 (51.4) | | 49 (52.7) | 50 (41.3) | | 27 (46.6) | 74 (47.4) | | 45 (56.3) | 136 (48.7) | | 88 (63.8) | 149 (40.9) | | 96 (52.7) | 343 (49.9) | | 175 (50.7) | 311 (44.9) | | 149 (43.1) |  |
| T3 | 6 (13.0) | 2 (8.7) | 13 (12.1) | 13 (24.1) | 20 (10.8) | | 31 (33.3) | 10 (8.3) | | 22 (37.9) | 27 (17.3) | | 22 (27.5) | 32 (11.5) | | 27 (19.6) | 57 (15.7) | | 32 (17.6) | 93 (13.5) | | 54 (15.7) | 87 (12.6) | | 47 (13.6) |  |
| Missing | 4 | 2 | 11 | 5 | 9 | | 4 | 11 | | 8 | 20 | | 8 | 29 | | 16 | 18 | | 9 | 63 | | 30 | 58 | | 29 |  |
| *p-value*^a^ |  | 0.852 |  | 0.102 |  | | <0.001 |  | | <0.001 |  | | 0.006 |  | | <0.001 |  | | 0.007 |  | | 0.523 |  | | 0.816 |  |
| *p-value*^b^ |  | 0.850 |  | 0.076 |  | | <0.001 |  | | <0.001 |  | | 0.009 |  | | <0.001 |  | | 0.008 |  | | 0.463 |  | | 0.856 |  |
| **Lymph nodal involvement (N) ^a^** |  |  |  |  |  | |  |  | |  |  | |  |  | |  |  | |  |  | |  |  | |  |  |
| N0 | 23 (50.0) | 17 (73.9) | 56 (50.9) | 28 (50.9) | 99 (53.2) | | 41 (44.1) | 58 (47.5) | | 22 (36.7) | 82 (51.2) | | 39 (49.4) | 142 (51.1) | | 53 (38.4) | 187 (52.2) | | 69 (38.1) | 338 (49.1) | | 142 (41.4) | 357 (51.6) | | 154 (44.3) |  |
| N+ | 23 (50.0) | 6 (26.1) | 54 (49.1) | 27 (49.1) | 87 (46.8) | | 52 (55.9) | 64 (52.5) | | 38 (63.3) | 78 (48.8) | | 40 (50.6) | 136 (48.9) | | 85 (61.6) | 171 (47.8) | | 112 (61.9) | 350 (50.9) | | 201 (58.6) | 335 (48.4) | | 194 (55.7) |  |
| Missing | 4 | 2 | 8 | 4 | 8 | | 4 | 10 | | 6 | 16 | | 9 | 30 | | 16 | 24 | | 10 | 62 | | 32 | 58 | | 27 |  |
| *p-value*^a^ |  | 0.058 |  | 1.000 |  | | 0.150 |  | | 0.165 |  | | 0.784 |  | | 0.015 |  | | 0.002 |  | | 0.019 |  | | 0.026 |  |
| *p-value*^b^ |  | 0.076 |  | 0.910 |  | | 0.162 |  | | 0.150 |  | | 0.776 |  | | 0.013 |  | | 0.002 |  | | 0.021 |  | | 0.027 |  |
| **Stage ^a^** |  |  |  |  |  | |  |  | |  |  | |  |  | |  |  | |  |  | |  |  | |  |  |
| Stage 0 + I | 14 (31.1) | 9 (39.1) | 18 (18.6) | 8 (16.7) | 49 (28.2) | | 7 (8.0) | 35 (32.7) | | 6 (10.7) | 35 (24.0) | | 8 (10.7) | 71 (26.8) | | 10 (7.7) | 97 (28.4) | | 30 (17.0) | 146 (23.0) | | 59 (18.5) | 180 (28.1) | | 78 (23.7) |  |
| Stage II | 11 (24.4) | 9 (39.1) | 45 (46.4) | 18 (37.5) | 55 (31.6) | | 31 (35.2) | 29 (27.1) | | 16 (28.6) | 48 (32.9) | | 30 (40.0) | 90 (34.0) | | 44 (33.8) | 123 (36.1) | | 51 (29.0) | 222 (35.0) | | 111 (34.8) | 222 (34.6) | | 114 (34.7) |  |
| Stage III + IV | 20 (44.4) | 5 (21.7) | 34 (35.1) | 22 (45.8) | 70 (40.2) | | 50 (56.8) | 43 (40.2) | | 34 (60.7) | 63 (43.2) | | 37 (49.3) | 104 (39.2) | | 76 (58.5) | 121 (35.5) | | 95 (54.0) | 266 (42.0) | | 149 (46.7) | 239 (37.3) | | 137 (41.6) |  |
| Missing | 5 | 2 | 21 | 11 | 20 | | 9 | 25 | | 10 | 30 | | 13 | 43 | | 24 | 41 | | 15 | 116 | | 56 | 109 | | 46 |  |
| *p-value*^a^ |  | 0.293 |  | 0.645 |  | | 0.002 |  | | 0.012 |  | | 0.023 |  | | <0.001 |  | | <0.001 |  | | 0.142 |  | | 0.444 |  |
| *p-value*^b^ |  | 0.205 |  | 0.384 |  | | 0.002 |  | | 0.009 |  | | 0.035 |  | | <0.001 |  | | <0.001 |  | | 0.088 |  | | 0.466 |  |
| **ER status ^a^** |  |  |  |  |  | |  |  | |  |  | |  |  | |  |  | |  |  | |  |  | |  |  |
| Negative | 9 (25.7) | 9 (40.9) | 24 (34.3) | 26 (66.7) | 39 (30.0) | | 29 (48.3) | 27 (34.2) | | 25 (64.1) | 40 (37.4) | | 30 (61.2) | 48 (26.8) | | 39 (47.0) | 60 (24.2) | | 37 (31.6) | 127 (26.3) | | 79 (33.1) | 134 (26.5) | | 74 (30.1) |  |
| Positive | 26 (74.3) | 13 (59.1) | 46 (65.7) | 13 (33.3) | 91 (70.0) | | 31 (51.7) | 52 (65.8) | | 14 (35.9) | 67 (62.6) | | 19 (38.8) | 131 (73.2) | | 44 (53.0) | 188 (75.8) | | 80 (68.4) | 355 (73.7) | | 160 (66.9) | 372 (73.5) | | 172 (69.9) |  |
| Missing | 15 | 3 | 48 | 20 | 64 | | 37 | 53 | | 27 | 69 | | 39 | 129 | | 71 | 134 | | 74 | 268 | | 136 | 244 | | 129 |  |
| *p-value*^a^ |  | 0.230 |  | 0.001 |  | | 0.014 |  | | 0.002 |  | | 0.005 |  | | 0.001 |  | | 0.134 |  | | 0.061 |  | | 0.301 |  |
| *p-value*^b^ |  | 0.342 |  | 0.007 |  | | 0.001 |  | | 0.010 |  | | <0.001 |  | | 0.027 |  | | 0.179 |  | | 0.061 |  | | 0.106 |  |
| **PR status ^a^** |  |  |  |  |  | |  |  | |  |  | |  |  | |  |  | |  |  | |  |  | |  |  |
| Negative | 13 (39.4) | 10 (45.5) | 30 (42.9) | 22 (56.4) | 50 (39.4) | | 34 (55.7) | 35 (44.9) | | 31 (79.5) | 47 (44.3) | | 36 (73.5) | 55 (31.6) | | 47 (58.8) | 74 (29.7) | | 55 (47.8) | 179 (37.7) | | 98 (42.1) | 201 (39.8) | | 111 (46.1) |  |
| Positive | 20 (60.6) | 12 (54.5) | 40 (57.1) | 17 (43.6) | 77 (60.6) | | 27 (44.3) | 43 (55.1) | | 8 (20.5) | 59 (55.7) | | 13 (26.5) | 119 (68.4) | | 33 (41.3) | 175 (70.3) | | 60 (52.2) | 296 (62.3) | | 135 (57.9) | 304 (60.2) | | 130 (53.9) |  |
| Missing | 17 | 3 | 48 | 20 | 67 | | 36 | 54 | | 27 | 70 | | 39 | 134 | | 74 | 133 | | 76 | 275 | | 142 | 245 | | 134 |  |
| *p-value*^a^ |  | 0.655 |  | 0.174 |  | | 0.035 |  | | <0.001 |  | | 0.001 |  | | <0.001 |  | | 0.001 |  | | 0.262 |  | | 0.105 |  |
| *p-value*^b^ |  | 0.705 |  | 0.334 |  | | 0.006 |  | | 0.001 |  | | <0.001 |  | | 0.002 |  | | 0.002 |  | | 0.223 |  | | 0.009 |  |
| **HER2 status ^a^** |  |  |  |  |  | |  |  | |  |  | |  |  | |  |  | |  |  | |  |  | |  |  |
| Negative | 22 (61.1) | 12 (75.0) | 39 (66.1) | 27 (84.4) | 75 (69.4) | | 31 (56.4) | 44 (73.3) | | 15 (48.4) | 65 (69.1) | | 33 (68.8) | 114 (72.6) | | 43 (53.8) | 182 (74.9) | | 82 (73.9) | 329 (80.2) | | 137 (66.5) | 310 (71.8) | | 156 (71.9) |  |
| Positive | 14 (38.9) | 4 (25.0) | 20 (33.9) | 5 (15.6) | 33 (30.6) | | 24 (43.6) | 16 (26.7) | | 16 (51.6) | 29 (30.9) | | 15 (31.3) | 43 (27.4) | | 37 (46.3) | 61 (25.1) | | 29 (26.1) | 81 (19.8) | | 69 (33.5) | 122 (28.2) | | 61 (28.1) |  |
| Missing | 14 | 9 | 59 | 27 | 86 | | 42 | 72 | | 35 | 82 | | 40 | 151 | | 74 | 139 | | 80 | 340 | | 169 | 318 | | 158 |  |
| *p-value*^a^ |  | 0.331 |  | 0.062 |  | | 0.098 |  | | 0.018 |  | | 0.961 |  | | 0.004 |  | | 0.837 |  | | <0.001 |  | | 0.972 |  |
| *p-value*^b^ |  | 0.443 |  | 0.061 |  | | 0.035 |  | | 0.007 |  | | 0.661 |  | | 0.003 |  | | 0.661 |  | | 0.003 |  | | 0.522 |  |
| **Grade ^a^** |  |  |  |  |  | |  |  | |  |  | |  |  | |  |  | |  |  | |  |  | |  |  |
| Grade I + II | 18 (52.9) | 7 (35.0) | 23 (41.1) | 5 (15.6) | 47 (40.5) | | 17 (30.4) | 31 (43.7) | | 10 (31.3) | 40 (37.4) | | 12 (27.9) | 68 (42.5) | | 21 (28.8) | 107 (45.0) | | 31 (29.5) | 206 (48.6) | | 83 (41.5) | 221 (50.0) | | 96 (43.8) |  |
| Grade III | 16 (47.1) | 13 (65.0) | 33 (58.9) | 27 (84.4) | 69 (59.5) | | 39 (69.6) | 40 (56.3) | | 22 (68.8) | 67 (62.6) | | 31 (72.1) | 92 (57.5) | | 52 (71.2) | 131 (55.0) | | 74 (70.5) | 218 (51.4) | | 117 (58.5) | 221 (50.0) | | 123 (56.2) |  |
| Missing | 16 | 5 | 62 | 27 | 78 | | 41 | 61 | | 34 | 69 | | 45 | 148 | | 81 | 144 | | 86 | 326 | | 175 | 308 | | 156 |  |
| *p-value*^a^ |  | 0.202 |  | 0.014 |  | | 0.196 |  | | 0.234 |  | | 0.270 |  | | 0.045 |  | | 0.007 |  | | 0.098 |  | | 0.135 |  |
| *p-value*^b^ |  | 0.233 |  | 0.161 |  | | 0.195 |  | | 0.251 |  | | 0.143 |  | | 0.164 |  | | 0.002 |  | | 0.235 |  | | 0.033 |  |
| **Surrogate subtype** |  |  |  |  |  | |  |  | |  |  | |  |  | |  |  | |  |  | |  |  | |  |  |
| Luminal A-like | 9 (36.0) | 5 (33.3) | 10 (26.3) | 5 (23.8) | 19 (23.5) | | 4 (12.1) | 13 (28.3) | | 1 (4.5) | 14 (22.6) | | 5 (16.7) | 39 (36.8) | | 4 (8.7) | 63 (37.3) | | 12 (17.9) | 117 (41.6) | | 38 (28.4) | 119 (36.2) | | 38 (25.3) |  |
| Luminal B-like | 3 (12.0) | 3 (20.0) | 10 (26.3) | 5 (23.8) | 24 (29.6) | | 5 (15.2) | 13 (28.3) | | 4 (18.2) | 15 (24.2) | | 4 (13.3) | 28 (26.4) | | 7 (15.2) | 46 (27.2) | | 28 (41.8) | 77 (27.4) | | 28 (20.9) | 70 (21.3) | | 48 (32.0) |  |
| Luminal HER2 positive | 6 (24.0) | 2 (13.3) | 11 (28.9) | 0 (0.0) | 14 (17.3) | | 13 (39.4) | 9 (19.6) | | 7 (31.8) | 14 (22.6) | | 4 (13.3) | 20 (18.9) | | 12 (26.1) | 27 (16.0) | | 9 (13.4) | 28 (10.0) | | 30 (22.4) | 67 (20.4) | | 36 (24.0) |  |
| HER2 positive | 2 (8.0) | 2 (13.3) | 3 (7.9) | 1 (4.8) | 6 (7.4) | | 5 (15.2) | 4 (8.7) | | 5 (22.7) | 7 (11.3) | | 5 (16.7) | 5 (4.7) | | 8 (17.4) | 9 (5.3) | | 3 (4.5) | 16 (5.7) | | 11 (8.2) | 20 (6.1) | | 4 (2.7) |  |
| TNBC | 5 (20.0) | 3 (20.0) | 4 (10.5) | 10 (47.6) | 18 (22.2) | | 6 (18.2) | 7 (15.2) | | 5 (22.7) | 12 (19.4) | | 12 (40.0) | 14 (13.2) | | 15 (32.6) | 24 (14.2) | | 15 (22.4) | 43 (15.3) | | 27 (20.1) | 53 (16.1) | | 24 (16.0) |  |
| Missing | 25 | 10 | 80 | 38 | 113 | | 64 | 86 | | 44 | 114 | | 58 | 202 | | 108 | 213 | | 124 | 469 | | 241 | 421 | | 225 |  |
| *p-value*^a^ |  | 0.875 |  | 0.007 |  | | 0.042 |  | | 0.081 |  | | 0.190 |  | | <0.001 |  | | 0.025 |  | | 0.001 |  | | 0.021 |  |
| *p-value*^b^ |  | 0.800 |  | 0.198 |  | | 0.018 |  | | 0.013 |  | | 0.004 |  | | 0.005 |  | | 0.040 |  | | 0.013 |  | | 0.020 |  |
| PrBC; Breast cancer diagnosed during pregnancy, PPBC; Breast cancer diagnosed post-delivery, comp.; Matched comparators, ER; oestrogen receptor, PR; progesterone receptor, HER2; human epidermal growth factor receptor 2  ^a^ Chi-squared test of association based on complete case data.  ^b^ Wald test based on imputed data. | | | | | | | | | | | | | | | | | | | | | | | | | | |

| **Supplemental Table S5. Treatment received by women diagnosed with breast cancer during pregnancy and 2 years post-delivery 1992-2018.** | | | | | | |
| --- | --- | --- | --- | --- | --- | --- |
|  | **During pregnancy** | | **1^st^ year post-delivery** | | **2^nd^ year post-delivery** | |
|  | **Matched**  **comparators**  **N (%)** | **PrBC**  **N (%)** | **Matched**  **comparators**  **N (%)** | **PPBC**  **N (%)** | **Matched**  **comparators**  **N (%)** | **PPBC**  **N (%)** |
| **Total no. of observations** | 362 | 181 | 998 | 499 | 1500 | 750 |
| **Surgery** |  |  |  |  |  |  |
| No/Other | 3 (0.9) | 4 (2.5) | 13 (1.5) | 6 (1.4) | 11 (0.8) | 7 (1.0) |
| Breast conserving surgery | 143 (43.2) | 50 (30.9) | 403 (45.0) | 138 (32.0) | 608 (44.8) | 256 (37.7) |
| Mastectomy/subcutaneous mastectomy | 185 (55.9) | 108 (66.7) | 479 (53.5) | 287 (66.6) | 737 (54.4) | 416 (61.3) |
| Missing | 31 | 19 | 103 | 68 | 144 | 71 |
| *p-value*^a^ |  | 0.016 |  | <0.001 |  | 0.009 |
| **Chemotherapy** |  |  |  |  |  |  |
| No | 63 (23.9) | 16 (12.7) | 200 (28.1) | 56 (16.3) | 300 (27.3) | 127 (23.6) |
| Yes | 201 (76.1) | 110 (87.3) | 512 (71.9) | 287 (83.7) | 800 (72.7) | 411 (76.4) |
| Missing | 98 | 55 | 286 | 156 | 400 | 212 |
| *p-value*^a^ |  | 0.010 |  | <0.001 |  | 0.112 |
| **Radiotherapy** |  |  |  |  |  |  |
| No | 101 (38.8) | 45 (36.6) | 220 (31.3) | 104 (30.6) | 376 (34.4) | 186 (35.0) |
| Yes | 159 (61.2) | 78 (63.4) | 484 (68.8) | 236 (69.4) | 716 (65.6) | 346 (65.0) |
| Missing | 102 | 58 | 294 | 159 | 408 | 218 |
| *p-value*^a^ |  | 0.671 |  | 0.829 |  | 0.833 |
| **Endocrine therapy** |  |  |  |  |  |  |
| No | 119 (46.7) | 67 (56.8) | 318 (46.6) | 186 (55.7) | 491 (46.3) | 244 (47.1) |
| Yes | 136 (53.3) | 51 (43.2) | 365 (53.4) | 148 (44.3) | 570 (53.7) | 274 (52.9) |
| Missing | 107 | 63 | 315 | 165 | 439 | 232 |
| *p-value*^a^ |  | 0.069 |  | 0.006 |  | 0.757 |
| **Anti-HER2 treatment** |  |  |  |  |  |  |
| No | 98 (64.5) | 49 (66.2) | 306 (73.0) | 127 (62.3) | 485 (76.7) | 231 (70.9) |
| Yes | 54 (35.5) | 25 (33.8) | 113 (27.0) | 77 (37.7) | 147 (23.3) | 95 (29.1) |
| Missing | 210 | 107 | 579 | 295 | 868 | 424 |
| *p-value*^a^ |  | 0.797 |  | 0.006 |  | 0.047 |
| PrBC; Breast cancer diagnosed during pregnancy, PPBC; Breast cancer diagnosed post-delivery  **^a^** Chi-squared test of association based on complete case data. | | | | | | |

| **Supplemental Table S6. Associations between pre- and post-delivery diagnosis and breast cancer death in women aged 18-44 years in Sweden between 1992-2018.** | | | | | |
| --- | --- | --- | --- | --- | --- |
|  | **Number of breast cancers** | **Number of deaths** | **Mortality rate per 1,000 person-years (95% CI)** | **Model 1 HR^a^ (95% CI)** | **Model 2 HR^a^ (95% CI)** |
| **Matched comparators** |  |  |  |  |  |
|  | 2860 | 528 | 19.1 (18.4-19.9) | 1.0 (ref) | 1.0 (ref) |
| **Women with PrBC & PPBC** | |  |  |  |  |
| PrBC – during pregnancy | 181 | 45 | 36.4 (27.2-48.7) | 1.5 (1.0-2.3) | 1.5 (1.0-2.3) |
| PPBC – 1^st^ year post-delivery | 499 | 133 | 39.4 (33.2-46.7) | 1.2 (0.9-1.5) | 1.1 (0.9-1.5) |
| PPBC – 2^nd^ year post-delivery | 750 | 156 | 28.3 (24.2-33.1) | 1.0 (0.8-1.3) | 1.0 (0.8-1.3) |
|  |  |  |  |  |  |
| PrBC - 1^st^ trimester | 25 | 3 | 18.6 (6.0-57.8) | 1.3 (0.4-4.5) | 1.2 (0.4-4.3) |
| PrBC - 2^nd^ trimester | 59 | 18 | 42.0 (26.5-66.7) | 1.8 (1.0-3.2) | 1.8 (1.0-3.2) |
| PrBC - 3^rd^ trimester | 97 | 24 | 37.1 (24.9-55.3) | 1.4 (0.8-2.3) | 1.4 (0.8-2.3) |
| PPBC - 0-6 months post-delivery | 154 | 39 | 37.8 (27.6-51.8) | 1.1 (0.8-1.6) | 1.1 (0.7-1.5) |
| PPBC - 7-12 months post-delivery | 345 | 94 | 40.0 (32.7-49.0) | 1.2 (0.9-1.6) | 1.2 (0.9-1.5) |
| PPBC - 13-18 months post-delivery | 375 | 79 | 28.2 (22.6-35.2) | 1.0 (0.8-1.3) | 1.0 (0.7-1.2) |
| PPBC - 19-24 months post-delivery | 375 | 77 | 28.3 (22.7-35.4) | 1.1 (0.9-1.5) | 1.1 (0.9-1.5) |
| PrBC; Breast cancer diagnosed during pregnancy, PPBC; Breast cancer diagnosed post-delivery, HR; Hazard rate Ratio.  ^a^ HR’s from Cox regression models (separate models were fitted for the broad and finer PABC exposure) on imputed datasets that were pooled using Rubin’s rules.  Model 1: adjusted for matching variables (age at diagnosis, year of diagnosis, quality register information availability), parity, country of birth, healthcare region, breast cancer subtypes, tumour stage.  Model 2: Same as Model 1 with further adjustment for surgery, chemotherapy, radiotherapy, endocrine therapy. | | | | | |
