# Supplementary material for: Worse histopathology and prognosis in women with breast cancer diagnosed during the second trimester of pregnancy
Source: ESMO Open. 2024 Mar 22;9(4):102972. doi: 10.1016/j.esmoop.2024.102972 (PMC10980937; doi:10.1016/j.esmoop.2024.102972)
Supplement: Supplementary Data [file mmc2.docx]

**Supplemental Methods**

**Details of multiple imputation**

We applied multiple imputation with chained equations (MICE) to account for missing information in important covariates (1). Multiple imputation is a two-step procedure involving the imputation step generating imputed datasets, and an analysis step where the analytical model is applied to each imputed dataset and the results are pooled together to combined estimates using Rubin’s rules.

**Preparation for imputation**

Table S1 lists the included variables in the imputation model (step 1) and the analytical model (step 2). Several variables were complete, while a few included missing data. The size of the dataset in the imputation analysis was N=15,171.

The variables that were imputed include: ER, PR, HER2, M stage, chemotherapy, radiotherapy, endocrine therapy, grade, tumour size T, nodal involvement N, and surgery type. Of the variables included in the imputation models, six were auxiliary variables that were not included in the analytical model: surgery type, chemotherapy, radiotherapy, endocrine therapy, event indicators for BCSS, and the Nelson-Aalen estimator for BCSS, as suggested by Falcaro et al (2).

**Supplemental Table S1.** Overview of variables included in the imputation model and analytical model

|  | **Missing among**  **N=15,167 (proportion missing)** | **Delete missing values from imputation analysis** | **Type in imputation**  **N=15,167** | **Imputation model** | **Analysis model** |
| --- | --- | --- | --- | --- | --- |
| Age | Complete |  | Complete | X | X |
| Year | Complete |  | Complete | X | X |
| Region | Complete |  | Complete | X | X |
| Mother’s country of birth | Complete |  | Complete | X | X |
| Quality register availability | Complete |  | Complete |  | X |
| Parity | Complete |  | Complete | X | X |
| PABC indicator | Complete |  | Complete | X | X |
| T | 1463/15171  (9.7%) |  | Imputed | X |  |
| N | 1468/15171  (9.7%) |  | Imputed | X |  |
| M | 2483/15171  (16.4%) |  | Imputed | X |  |
| Stage | 2603/15171  (17.2%) |  |  |  | X |
| Nottingham grade | 6946/15171  (45.8%) |  | Imputed | X |  |
| ER | 5372/15171 (35.4%) |  | Imputed | X |  |
| PR | 5464/15171 (36.0%) |  | Imputed | X |  |
| HER2 | 8764/15171 (57.8%) |  |  |  |  |
| HER2 after deterministic prediction | 7726/15171 (50.9%) |  | Imputed | X |  |
| Subtype | 9736/15171 (64.2%) |  |  |  | X |
| Surgery (set None/Other to missing) | 1845/15171  (12.2%) |  | Auxiliary | X |  |
| Chemotherapy | 4432/15171  (29.2%) |  | Auxiliary | X |  |
| Radiotherapy | 4499/15171  (29.7%) |  | Auxiliary | X |  |
| Endocrine therapy | 4847/15171  (32.0%) |  | Auxiliary | X |  |
| dead_BC, cumhaz | Complete |  | Auxiliary | X |  |

PABC=Pregnancy-Associated Cancer

dead_BC=event indicator for BCSS

cumhaz=Nelson-Aalen estimator for BCSS

**Step 1: Imputation model**

We used Stata mi package including command mi impute (Stata/BE 17.0). A total of *m*=61 imputed datasets, with 50 iterations, were generated. The number of imputed datasets *m* was chosen to be larger than the highest percentage of missing values among the variables (HER2=57.8%), according to the rule of thumb by White and colleagues (2011) (1). The HER2 variable was deterministically predicted from targeted therapy reception before it was included in the imputation model. In the chained equations, logistic regression models were applied to binary outcome variables (ER, PR, HER2, M stage, chemotherapy, radiotherapy, endocrine therapy), while multinomial regression was used for categorical outcome variables (grade, tumour size T, nodal involvement N, surgery type). The imputation model included the variables listed in Table S1.

**Step 2: Analysis model and pooling**

To estimate pooled hazard ratios for BCSS, we used Stata mi estimate command, which applies Rubin’s rules to the imputed datasets. We combined the ER, PR, HER2 and grade into a subtype variable using mi passive: utility in Stata. The analytical Cox model included the variables listed in Table S1, in addition to other already complete variables.

In a separate step, we estimated frequencies and proportions of tumour characteristics across pre- and post-delivery intervals based on logistic regression models for binary outcomes and multinomial logistic regression for categorical outcomes. In addition, we estimated crude mortality rates from the multiple imputed datasets and calculated the pooled estimates (crude probabilities and standard error) by applying Rubin’s rules ourselves (1).
